# Proteomic Discovery of Biomarkers to Predict Prognosis of High-Grade Serous Ovarian Carcinoma

**DOI:** 10.3390/cancers12040790

**Published:** 2020-03-26

**Authors:** Se Ik Kim, Minsun Jung, Kisoon Dan, Sungyoung Lee, Cheol Lee, Hee Seung Kim, Hyun Hoon Chung, Jae-Weon Kim, Noh Hyun Park, Yong-Sang Song, Dohyun Han, Maria Lee

**Affiliations:** 1 Department of Obstetrics and Gynecology, Seoul National University College of Medicine, Seoul 03080, Korea; seikky@naver.com (S.I.K.); bboddi0311@gmail.com (H.S.K.); chhkmj@gmail.com (H.H.C.); kjwksh@snu.ac.kr (J.-W.K.); pnhkhr@snu.ac.kr (N.H.P.); yssong@snu.ac.kr (Y.-S.S.); 2 Department of Pathology, Seoul National University College of Medicine, Seoul 03080, Korea; jjunglammy@gmail.com (M.J.); fejhh@hanmail.net (C.L.); 3 Proteomics Core Facility, Biomedical Research Institute, Seoul National University Hospital, Seoul 03082, Korea; kisoona@snuh.org; 4 Center for Precision Medicine, Seoul National University Hospital, Seoul 03080, Korea; biznok@snu.ac.kr

**Keywords:** ovarian neoplasms, high-grade serous carcinoma, proteomics, immunohistochemistry, prognosis

## Abstract

Initial identification of biomarkers predicting the exact prognosis of high-grade serous ovarian carcinoma (HGSOC) is important in precision cancer medicine. This study aimed to investigate prognostic biomarkers of HGSOC through proteomic analysis. We conducted label-free liquid chromatography-mass spectrometry using chemotherapy-naïve, fresh-frozen primary HGSOC specimens, and compared the results between a favorable prognosis group (progression-free survival (PFS) ≥ 18 months, *n* = 6) and a poor prognosis group (PFS < 18 months, *n* = 6). Among 658 differentially expressed proteins, 288 proteins were upregulated in the favorable prognosis group and 370 proteins were upregulated in the poor prognosis group. Using hierarchical clustering, we selected α1-antitrypsin (AAT), nuclear factor-κB (NFKB), phosphomevalonate kinase (PMVK), vascular adhesion protein 1 (VAP1), fatty acid-binding protein 4 (FABP4), platelet factor 4 (PF4), apolipoprotein A1 (APOA1), and α1-acid glycoprotein (AGP) for further validation via immunohistochemical (IHC) staining in an independent set of chemotherapy-naïve primary HGSOC samples (*n* = 107). Survival analyses revealed that high expression of AAT, NFKB, and PMVK were independent biomarkers for favorable PFS. Conversely, high expression of VAP1, FABP4, and PF4 were identified as independent biomarkers for poor PFS. Furthermore, we constructed models predicting the 18-month PFS by combining clinical variables and IHC results. Through leave-one-out cross-validation, the optimal model was based on initial serum CA-125, germline *BRCA1/2* mutations, residual tumors after surgery, International Federation of Gynecology and Obstetrics (FIGO) stage, and expression levels of the six proteins. The present results elucidate the proteomic landscape of HGSOC and six protein biomarkers to predict the prognosis of HGSOC.

## 1. Introduction

Ovarian cancer, one of the most fatal gynecologic malignancies, is a global burden with 295,414 new cases and 184,799 deaths estimated each year [1]. Ovarian cancer is the fifth leading cause of female cancer-related deaths in the United States [2]. The predominant histologic type is high-grade serous ovarian carcinoma (HGSOC) [3], for which aggressive cytoreductive surgery followed by taxane- and platinum-based chemotherapy is an established standard of care [4,5]. Although the initial response rate is high, patients with HGSOC, especially those at advanced stages, eventually experience relapse [6]. In the era of precision medicine, it is important to initially identify biomarkers to accurately predict the exact prognosis of HGSOC to facilitate personalized treatment.

Mass spectrometry (MS)-based proteomics has been widely used to characterize molecular components and underlying mechanisms associated with various malignancies such as colorectal [7], breast [8], lung [9], and ovarian cancers [10,11,12]. Currently, this emerging technology is used for high-throughput analysis for simultaneous quantification of numerous proteins and discovery of prognostic biomarkers in individual samples. The Clinical Proteomic Tumor Analysis Consortium (CPTAC) provides proteogenomic insights into HGSOC by performing extensive proteomic profiling and correlating results with data contained in The Cancer Genome Atlas (TCGA) database [10]. However, these biomarkers need to be subjected to specific validation studies before being clinically applied.

In this study, we performed label-free quantitative proteomic analysis of chemotherapy-naïve, fresh-frozen primary ovarian cancer tissues to elucidate prognostic protein biomarkers of HGSOC. We then validated our findings via immunohistochemical (IHC) staining in an independent dataset.

## 2. Results

### 2.1. Patient Characteristics in the Proteomic Analysis

Clinicopathologic characteristics of 12 patients with HGSOC, for whom proteomic analysis was performed, are presented in Table S1. Mean patient age was 56.5 years (range 42.0–74.1 years). No differences in patient age, menopausal status, or family history of breast cancer were observed between the good and poor prognosis groups. Initial serum levels of CA-125, International Federation of Gynecology and Obstetrics (FIGO) stage, and residual tumor after primary debulking surgery (PDS) were also similar between the two groups. Of the 12 patients, 4 and 2 patients harbored germline *BRCA1* and *BRCA2* mutations, respectively, while the other 6 patients harbored wild-type *BRCA1/2*. The median length of observations was 58.1 months, during which 11 patients (91.7%) experienced disease recurrence. Patients in the good prognosis group had significantly better progression-free survival (PFS) than those in the poor prognosis group (median, 26.0 vs. 16.9 months; *p* = 0.001) (Figure S1).

### 2.2. Results of Proteomic and Bioinformatic Analyses

#### 2.2.1. Global Proteomic Analysis of Ovarian Cancer Tissues

**Figure 1:**
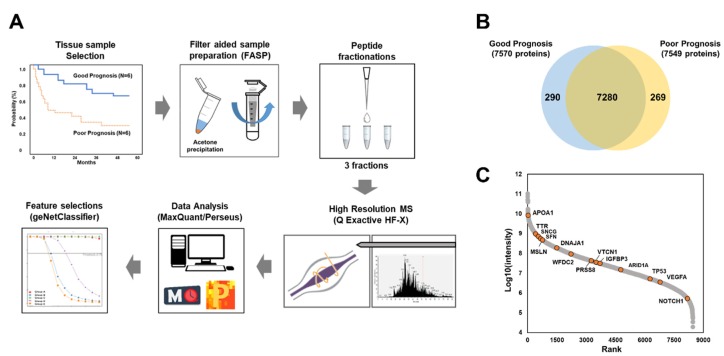
Proteomic analysis of ovarian cancer tissues with respect to survival outcome: (**A**) the protocol for proteomic analysis; (**B**) total number of proteins identified in each group of samples; (**C**) dynamic range of candidate biomarkers for high-grade serous ovarian carcinoma. MS, mass spectrometry.

To identity prognostic biomarkers for HGSOC, we performed MS-based label-free quantification using chemotherapy-naïve, fresh-frozen cancer tissues resected from the primary (non-metastatic) ovarian mass intraoperatively during the debulking surgery (*n* = 12); the good and poor prognosis groups contained six patients per group (Figure 1A). To expand the coverage of the identified ovarian proteome, we used pooled samples to generate a spectral library including 8520 protein groups corresponding to 93,355 unique peptides (Table S2). In the individual samples, 7839 protein groups were identified at a false discovery rate (FDR) of 1%. On average, 5900 protein groups were quantified per sample (Figure 1B). Signal intensities for total quantified proteins spanned approximately seven orders of magnitudes (Figure 1C), with several well-known ovarian cancer markers, such as apolipoprotein A1 (APOA1), transthyretin (TTR), synuclein gamma (SNCG), stratifin (SFN), mesothelin (MSLN), DnaJ heat shock protein family (Hsp40) member A1 (DNAJA1), WAP four-disulfide core domain 2 (WFDC2), serine protease 8 (PRSS8), V-set domain containing T cell activation inhibitor 1 (VTCN1), insulin like growth factor binding protein 3 (IGFBP3), AT-rich interaction domain 1A (ARID1A), tumor protein p53 (TP53), vascular endothelial growth factor A (VEGFA), and notch receptor 1 (NOTCH1), being identified [13]. Pearson’s correlation coefficients were correspondingly high at 0.82–0.84 for inter- and intra-tissue replicates (Figure S2).

On assessing tumor purity, using the ESTIMATE tool in the R package, mean (± standard deviation) tumor purity score across the samples was 80.0 (± 1.7) and 74.8 (± 3.0) in the good and poor prognosis groups, respectively, indicating that the tumor purity was adequate to distinguish the tumor’s signal from those of other cells (Figure S3) [14].

#### 2.2.2. Label-Free Quantification

**Figure 2:**
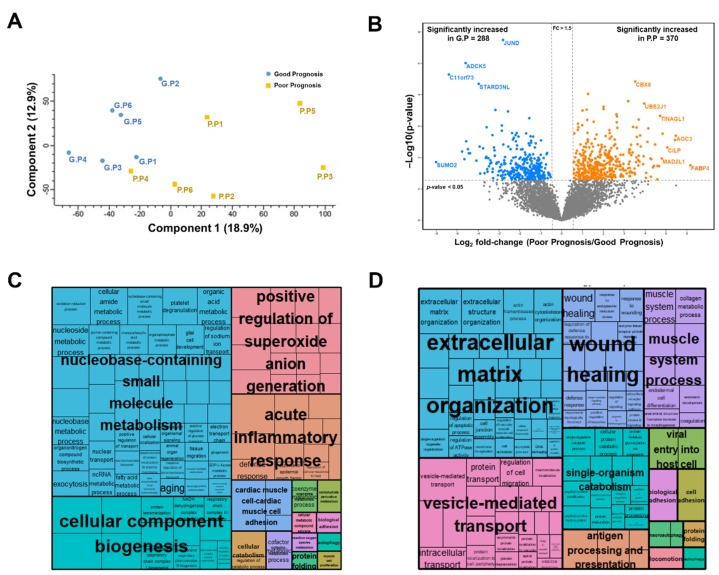
Statistical and functional differences between good and poor prognosis groups: (**A**) principal component analysis (PCA); (**B**) volcano plot; (**C**) gene ontology biological process (GOBP) enrichment tree-map of upregulated proteins in the good prognosis group; (**D**) GOBP enrichment tree-map of upregulated proteins in the poor prognosis group.

Furthermore, we compared the good and poor prognosis groups via principal component analysis (PCA) of a filtered list with approximately 6128 proteins (with 70% of valid intensity-based absolute quantification (iBAQ) values in at least one group). Although tumor proteomes correlated regardless of prognosis (Figure S2), the two groups were independently separated (Figure 2A).

To obtain functional insights into the proteomic data, we constructed a volcano plot to compare the expression levels between the good and poor prognosis groups. Pairwise comparisons via a *t*-test and filtering (*p* < 0.05; fold-change, > 1.5) revealed significant alterations in 658 proteins, of which 370 were upregulated and 288 were downregulated in the poor prognosis group (Figure 2B and Table S3). Gene ontology (GO) enrichment analysis revealed that proteins upregulated in the good prognosis group were significantly enriched for terms such as “nucleobase-containing small molecule metabolism”, ”positive regulation of superoxide anion generation”, “acute inflammatory response”, “cellular component biogenesis”, and “autophagy” (Figure 2C and Table S4). In contrast, proteins upregulated in the poor prognosis group were significantly enriched in ”extracellular matrix organization”, ”wound healing”, ”muscle system process”, “vesicle-mediated transport”, “single-organism catabolism”, and “antigen processing and presentation“ (Figure 2D and Table S4).

#### 2.2.3. Selection of Candidate Prognostic Biomarkers

**Figure 3:**
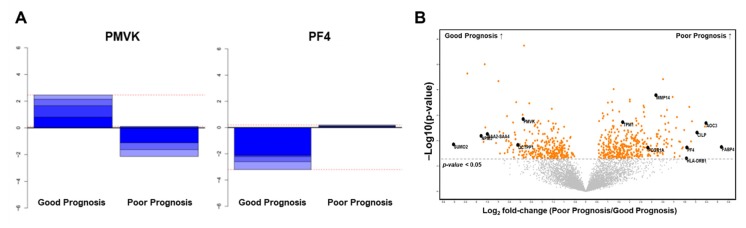
Selection of prognostic biomarker candidates using unbiased machine learning: (**A**) Discriminant power plots of the up-regulated gene (phosphomevalonate kinase, PMVK) in the good prognosis group and the up-regulated gene (platelet factor 4, PF4) in the poor prognosis group; (**B**) volcano plot with the 13 statistically significant differentially expressed proteins.

Further, we analyzed our proteomic data using the R/Bioconductor package “geNetClassifier (GNC)” [15] to rank proteins with the greatest discriminant power in an unbiased manner (Figure 3A). In total, 229 proteins exceeded the posterior probability cut-off (>0.95) and were used in training the support vector machine (Table S5). The lowest error rate achieved by GNC was 0.08 (8%) and corresponded to 41 proteins.

Among the top 20 proteins ranked through support-vector-machine analysis, 13 overlapped with significantly differentially expressed proteins (DEPs) (Figure 3B). After reviewing the data of each protein in the Human Protein Atlas database (https://www.proteinatlas.org) and evaluating the association between its expression and survival outcome in various malignancies, we selected phosphomevalonate kinase (PMVK), vascular adhesion protein 1 (VAP1), fatty acid-binding protein 4 (FABP4), and platelet factor 4 (PF4) as our biomarker candidates.

We further selected prognostic biomarker candidates on the basis of the following parameters: (1) significantly differentially expressed between the two groups, revealed through the *t*-test (*p* < 0.05); (2) quantification of expression in all samples; (3) presence of survival outcome data based on their expression in other malignancies; (4) availability of a commercial antibody; and (5) potential clinical utility (e.g., probability of being found in the blood). Thus, α1-antitrypsin (AAT), nuclear factor-κB (NFKB), APOA1, and α1-acid glycoprotein (AGP) were selected as additional biomarker candidates.

### 2.3. Validation of Protein Biomarkers through IHC Analysis

Prognostic validation of protein biomarkers was performed using tissue specimens obtained from 107 HGSOC patients, including the 12 patients whose tissue specimens were subjected to proteomic analysis. Clinicopathologic characteristics of 107 patients with HGSOC are presented in Table 1. The percentage of advanced-stage (FIGO III/IV) disease was 91.6% (98/107). Of these 107 patients, 102 (95.3%) underwent PDS, while 5 (4.7%) underwent neoadjuvant chemotherapy (NAC) followed by interval debulking surgery. Optimal debulking (no gross residual tumor) was achieved in 68.2% (73/107). Germline *BRCA1/2* mutations were observed in 50.5% (54/107). Median length of observation was 23.7 months; during this observation, 51 patients (47.7%) experienced relapse. The median PFS was 26.0 months, and the three-year PFS rate was 22.2%.

Herein, we used chemotherapy-naïve, formalin-fixed paraffin-embedded (FFPE) cancer tissues cut from the primary (non-metastatic) ovarian mass (*n* = 107). For the 12 patients, the specimens were the same as those used for proteomic analysis. Among the DEPs, AAT, NFKB, PMVK, VAP1, FABP4, PF4, APOA1, and AGP were subjected to further prognostic validation through IHC staining. Most tumor cells presented cytoplasmic and/or membranous staining patterns, except for VAP1, FABP4, PF4, and AGP, which occasionally displayed focal nuclear staining. APOA1 and AGP were also detected in the fibrotic stroma and inflammatory cells (Figure S4).

**Figure 4:**
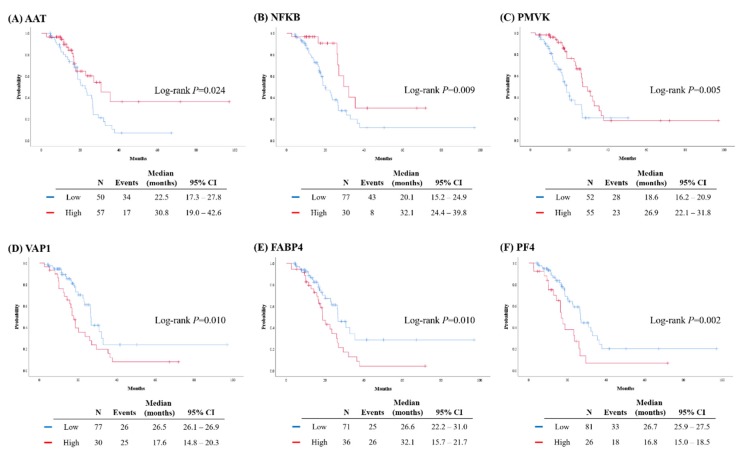
Comparison of progression-free survival (PFS) based on the expression levels of proteins: (**A**) α1-antitrypsin (AAT); (**B**) nuclear factor-κB (NFKB); (**C**) PMVK; (**D**) vascular adhesion protein 1(VAP1); (**E**) fatty acid-binding protein 4 (FABP4); (**F**) PF4.

We then compared patient survival outcomes with respect to the expression levels of each protein and observed significant differences in PFS for the six proteins as follows: the group of patients with high expression levels of AAT, NFKB, and PMVK presented better PFS than those with low expression levels (*p* = 0.024, *p* = 0.009, and *p* = 0.005, respectively). For VAP1, FABP4, and PF4, the high expression group presented a reduced PFS relative to the low expression group (*p* = 0.010, *p* = 0.010, and *p* = 0.002, respectively). However, the PFS did not significantly differ between patients with high and low expression levels of APOA1 and AGP, as determined through IHC staining (Figure 4).

We further compared the clinicopathologic characteristics of patients with high and low expression levels of the six relevant protein biomarkers. No differences in expression levels were observed with respect to patient age (≥55 vs. <55 years), FIGO stage (I–II vs. III–IV), or germline *BRCA1/2* mutational status (mutation vs. wild-type) between the high and low expression groups. Patients with high NFKB expression levels achieved no gross residual tumor after surgery more commonly than those with low NFKB expression levels (86.7% vs. 61.0%; *p* = 0.020). On assessing the platinum sensitivity of patients with respect to the expression profile of each protein, we observed a significant difference only for PVMK; patients with high PVMK expression levels were more sensitive to platinum-based chemotherapy than those with low PMVK expression levels (95.3% vs. 75.6%; *p* = 0.023) (Table S6).

On multivariate analyses adjusting patient age at diagnosis, initial serum CA-125 level, FIGO stage, residual tumor after surgery, and germline *BRCA1/2* mutational status, high expression of AAT, NFKB, and PMVK were identified as independent favorable prognostic biomarkers for PFS (AAT (adjusted hazard ratio (aHR), 0.398; 95% confidence interval (CI), 0.207–0.768; *p* = 0.006), NFKB (aHR, 0.424; 95% CI, 0.196–0.920; *p* = 0.030), and PMVK (aHR, 0.430; 95% CI, 0.228–0.809; *p* = 0.009)). In contrast, high expression of VAP1, FABP4, and PF4 were considered independent poor prognostic biomarkers for PFS (VAP1 (aHR, 1.911; 95% CI, 1.089–3.354; *p* = 0.024), FABP4 (aHR, 1.908; 95% CI, 1.093–3.331; *p* = 0.023), and PF4 (aHR, 2.071; 95% CI, 1.139–3.765; *p* = 0.017)) (Table 2).

Furthermore, we constructed models predicting 18-month PFS by combining clinical variables and IHC results. Here, we considered two types of models: regression- and score-based. The performance of predictive models was evaluated via the leave-one-out cross validation method for the regression-based models. The best model included the following variables: initial serum CA-125 levels (≥700 vs. <700 IU/mL), germline *BRCA1/2* mutational status (mutation vs. wild-type), residual tumor after surgery (gross vs. no gross), FIGO stage (III–IV vs. I–II), and expression levels (high or low) of the six protein biomarkers on IHC staining of ovarian cancer tissue. For the regression-based model, the estimated areas under the receiver operating characteristic curves (AUCs) from the training and test datasets were 0.898 and 0.776, respectively. In the score-based model, the scores of each predictor (0 or 1) were added to give a total score. Samples with the total score ≥7 were classified as “high-risk”, and showed an AUC of 0.855. Both regression- and score-based models displayed better performance than those comprising only clinical variables (Table S7).

## 3. Discussion

In the current study, we performed label-free liquid chromatography-mass spectrometry (LC-MS/MS)-based proteomic analysis on chemotherapy-naïve, fresh-frozen primary HGSOC tissues. Upon validation with complementary IHC staining for FFPE HGSOC tissue specimens, we identified six protein biomarkers to predict the prognosis of HGSOC; expression levels of AAT, NFKB, PMVK, VAP1, FABP4, and PF4 in ovarian cancer tissue were associated with PFS.

AAT, encoded by *SERPINA1* in humans, is a serine protease inhibitor that influences tumor behavior depending on the context and/or cancer type. Consistent with our results, enrichment of *SERPINA1* mRNA was associated with a good prognosis in HGSOC [16]; however, no association was observed between survival of patients with HGSOC and AAT expression levels, as assessed via IHC staining [17]. Two previous studies evaluated serum AAT levels in patients with epithelial ovarian cancer and reported that AAT may contribute to a differential diagnosis and help predict chemoresistance [18,19].

NFKB is constitutively activated in several cancers. The p100 subunit of NFKB, a precursor of the active p52 subunit, has been suggested to counteract the tumorigenic effects of p52 in breast cancer [20]. In ovarian cancer, NFKB p52 promoted cancer progression, resulting in an unfavorable prognosis [21]. To our knowledge, our study is the first to report the association between high expression of NFKB p100 and improved PFS in patients with HGSOC.

Furthermore, this study shows that PMVK, an enzyme involved in cholesterol synthesis and lipid metabolism, can be considered a novel prognostic biomarker for HGSOC. In estrogen receptor-positive breast cancer, high expression of *PMVK* gene was positively associated with responses to chemotherapeutic agents [22]. Similarly, herein, high expression of PMVK, assessed via IHC staining, was significantly associated with platinum sensitivity and improved the survival of patients with HGSOC.

VAP1, an adhesion molecule mediating interactions between various inflammatory and endothelial cells, may be associated with tumor invasion and metastasis. High expression of VAP1 was associated with a lower overall survival in breast cancer [23,24]. In our study, VAP was identified as a poor prognostic factor for PFS in patients with HGSOC.

A previous study reported that FABP4 promotes HGSOC progression by mediating lipid metabolism in cancer cells [25]. Furthermore, the present results indicate that high expression of FABP4, assessed via IHC staining, was associated with a poor PFS.

PF4 is a platelet-activating chemokine that induces thrombocytosis and thromboembolism. High serum PF4 levels were associated with poor survival and an increased risk of venous thromboembolism in patients with pancreatic adenocarcinoma [26]. According to a microarray study using 51 HGSOC samples, increased expression of PF4 mRNA was negatively associated with patients’ overall survival [27]. Similarly, our study results also indicate that high expression of PF4, assessed via IHC staining, was associated with a reduced PFS.

APOA1 and AGP are acute-phase reactants, and their serum levels have been assessed in epithelial ovarian cancer to assess their potential as diagnostic or prognostic predictive biomarkers [28,29,30,31]. However, tissue expression and clinical implications of APOA1 and AGP in HGSOC have not been determined. In the current study, we revealed that expressions of APOA1 and AGP in HGSOC cancer tissues were not associated with patient survival outcomes. Furthermore, in several tissue microarray (TMA) cores, APOA1 and AGP were expressed strongly only in the stroma and inflammatory cells, but not in cancer cells, suggesting the host immune response to cancer.

A potential limitation of our study is that the menopausal status may have influenced the expression levels of AAT, NFKB, PMVK, VAP1, FABP4, and PF4. However, during the proteomic analysis, the proportion of menopausal women was the same between the good and poor prognosis groups (66.7%). Moreover, during subsequent IHC analysis for prognostic validation, the patients’ ages were adjusted for multivariate analyses to identify the prognostic factors for PFS.

Since the CPTAC presented a proteomic landscape of 169 HGSOC tumors [10], several studies have focused on HGSOC using MS-based proteomics. Coscia et al. performed integrative proteomic profiling of ovarian cancer cell lines and HGSOC tumors and revealed two distinct clusters, epithelial and mesenchymal, which displayed different clinical outcomes [11]. Dieters-Castator et al. performed label-free quantitative proteomic analysis using 10 fresh-frozen HGSOC tissues and 10 fresh-frozen endometrioid carcinoma tissues, and identified diagnostic biomarkers specific to endometrioid carcinoma. Furthermore, the eight-marker panel, generated in that study, showed good performance in discriminating endometrioid carcinoma from HGSOC [12].

Unlike previous studies focused on a differential diagnosis or clustering of HGSOC, this study aimed to investigate protein biomarkers predicting survival outcomes. IHC staining on FFPE HGSOC tissue specimens revealed that expression levels of the six protein biomarkers were not different between the early-stage and advanced-stage disease, and between diseases with high and low initial serum CA-125 levels. Nevertheless, our results indicate that high expressions of AAT, NFKB, and PMVK are favorable prognostic biomarkers for PFS, whereas high expressions of VAP1, FABP4, and PF4 are poor prognostic biomarkers for PFS. These six protein biomarkers, along with well-known prognostic factors including the stage and size of residual tumors, are expected to increase the accuracy of predicting relapse after primary treatment. Such improvements in prognostic prediction would facilitate the development of individualized therapies. For instance, if a patient is identified as being at high risk for recurrence, she may receive intraperitoneal chemotherapy or bevacizumab maintenance therapy in addition to the standard treatment and would be placed on a frequent surveillance schedule for earlier detection of relapse. Moreover, each of the six protein biomarkers, identified in our present study, can be considered a target for novel molecular therapeutic agents against HGSOC. However, further translational studies using cell lines and cancer tissues are essential for assessing biological effectiveness of the protein biomarkers and for identifying relevant pathways.

Herein, we performed IHC staining to validate candidate prognostic protein biomarkers. IHC is more cost-effective and simpler to use in the clinical setting than DNA or RNA PCRs and exome- or transcriptome-level next-generation sequencing methods. IHC can be utilized for prognosis prediction immediately during pathologic examination of the tissue obtained during ovarian cancer surgery.

For further studies validating a predictive model consisting of all the six protein biomarkers, we calculated the estimated powers for various sample sizes and censoring rates, which were drawn from a simple simulation study (*p* < 0.05; 1000 replicates), using the estimates from our multivariate model and the predictor frequencies from our dataset (Figure S5).

This study has several limitations. First, owing to the retrospective study design, issues such as selection bias might exist. Second, the sample size used in our study may be insufficient for further discovery and validation of protein biomarkers. Third, external validation of our study results is necessary. Fourth, additional studies, elucidating the mechanisms of action of each protein biomarker, were not performed herein. Lastly, we did not evaluate interactions between among these protein biomarkers. Despite these limitations, we faithfully applied a two-step approach consisting of proteomic and bioinformatic analyses and subsequent IHC staining for HGSOC tissue to identify prognostic protein biomarkers. Moreover, we developed predictive models comprising the six protein biomarkers and clinical variables for 18-month PFS of HGSOC patients. Such models displayed better prediction potential than those comprising only clinical variables. Especially, the proposed predictive model was further simplified as a score-based model, which provides comparable performance and substantial intuitiveness.

## 4. Materials and Methods

This retrospective study was approved by the Institutional Review Board of Seoul National University Hospital (SNUH; No. C-1712-083-907), and conducted in accordance with the Declaration of Helsinki.

### 4.1. Study Design

This study included two steps: (1) proteomic and bioinformatic analysis for biomarker discovery; and (2) IHC staining for prognostic validation of candidate biomarkers (Figure S6).

In the first step, we used fresh-frozen primary (non-metastatic) ovarian cancer tissues obtained intraoperatively and stored at the SNUH Human Biobank for research purposes. We identified patients who met the following inclusion criteria: (1) older than 18 years; (2) diagnosed with HGSOC between June 2012 and December 2016; (3) underwent PDS; and (4) agreed to donate biospecimens and provide written informed consent. Patients with any malignancy other than ovarian cancer, those who received NAC, those with insufficient clinical data or those lost to follow-up, or those with severe co-morbidities were excluded. On the basis of their PFS, patients were divided into the favorable (good) prognosis group (≥18 months) and poor prognosis group (<18 months). In total, 12 patients from the two groups (6 for each group) were selected for further proteomic analysis, and proteomic profiles were compared between the two groups.

In the second step, we used FFPE primary (non-metastatic) ovarian cancer tissues stored in the pathology archive of SNUH. In contrast with the first step, we identified patients meeting the following inclusion criteria: (1) older than 18 years; (2) diagnosed with HGSOC between June 2012 and December 2018; (3) whose ovarian cancer tissue was obtained during chemotherapy-naïve status (such as at the time of PDS, or during diagnostic laparoscopy in case of NAC); and (4) agreed to donate their pathologic specimens for research purposes and provided written informed consent. Patients with insufficient clinical data or those lost to follow-up or those with severe co-morbidities were excluded. In total, 107 patients with primary HGSOC were included in this step.

### 4.2. Proteomic and Bioinformatic Analyses

#### 4.2.1. Tissue Preparation

Tissue samples were prepared using filter-aided sample preparation (FASP), as previously described [32]. Briefly, frozen tissue samples were homogenized using lysis buffer (4% sodium dodecyl sulfate (SDS), 2 mM Tris(2-carboxyethyl)phosphine (TCEP), and 0.1 M Tris-HCl pH 7.4), and protein concentration was determined using a reducing agent-compatible bicinchoninic acid (BCA) protein assay kit (Thermo Fisher Scientific, Waltham, MA, USA) in accordance with the manufacturer’s instructions. To eliminate contaminants, we performed acetone precipitation using 250 µg of the lysate at –20 °C. Each protein pellet was dissolved in 50 µL reduction buffer (4% SDS, 0.1 mM dithiothreitol (DTT), and 0.1 M Tris-HCl, pH 7.4) and heated at 95 °C for 15 min. The reduced proteins were loaded onto a 30 K spin filter (Millipore, Billerica, MA, USA), and buffer was exchanged for UA solution (8 M urea in 0.1 M Tris-HCl, pH 8.5) via centrifugation. After triple UA exchanging, the reduced cysteines were alkylated with 0.05 M iodoacetamide (IAA) in UA solution for 30 min at ambient temperature in the dark. Thereafter, UA buffer was exchanged for 40 mM ammonium bicarbonate (ABC), and the samples were digested with trypsin (enzyme to substrate ratio of 1:100) at 37 °C for 16 h. Further, the digested peptides were harvested via centrifugation, and an additional elution step was performed using 40 mM ABC and 0.5 M NaCl.

#### 4.2.2. Desalting and Peptide Fractionation of Individual Samples

Peptide concentrations were measured using the tryptophan fluorescence (WF) assay, as previously described [33]. Digested peptides (20 μg) were acidified with trifluoroacetic acid (TFA) and then loaded directly onto house-made Stage-Tip with polystyrenedivinylbenzene-reversed phase sulfonate (SDB-RPS) material [34]. StageTip was washed thrice with 100 μL 0.2% TFA. Three fractionations were performed using elution buffer 1, 2, and 3. All eluted peptides were dried in a SpeedVac centrifuge.

#### 4.2.3. Offline High-pH Reversed-Peptide Fractionation for Library Construction

For library construction, pooled peptides were fractionated via high pH reversed-phase liquid chromatography (RPLC) using an Agilent 1290 bioinert high performance liquid chromatography (HPLC) (Agilent, Santa Clara, CA, USA) equipped with an analytical column (4.6 × 250 mm, 5 μm), as previously described [32]. Solvent A consisted of 15 mM ammonium hydroxide in water, and solvent B consisted of 15 mM ammonium hydroxide in 90% acetonitrile (ACN). The peptides were separated along a gradient of 5%–35% ACN at a flow rate of 0.2 mL/min. In total, 96 fractions were concatenated to mix different parts of the gradient into 24 fractions. The fractions were lyophilized and stored at −80 °C until MS analysis.

#### 4.2.4. LC-MS/MS Analysis

All LC-MS/MS analyses were conducted using an Ultimate 3000 UHPLC system (Dionex, Sunnyvale, CA, USA) coupled with Q-Exactive HF-X mass spectrometry (Thermo Fisher Scientific, Waltham, MA, USA), as previously described with some modifications [32]. Peptides were separated on a two-column system equipped with a trap column (300 µm × 5 mm) and an analytic column (75 µm × 50 cm), using 90-min gradients from 7% to 32% ACN at a flow rate of 300 nl/min. Column temperature was maintained at 60 °C using a column heater. For label-free quantification using the data-dependent acquisition (DDA) method, a survey scan (350 to 1650 *m*/*z*) was acquired with a resolution of 70,000 at *m*/*z* 200. A top-15 method was used to select the precursor ion with an isolation window of 1.2 *m*/*z*. MS/MS spectra were acquired at a higher-energy collisional dissociation (HCD)-normalized collision energy (NCE) of 30 with a resolution of 17,500 at *m*/*z* 200. Maximum ion injection durations for the full and MS/MS scans were 20 and 100 ms, respectively.

#### 4.2.5. Data Processing

All MS raw files were processed using MaxQuant (version 1.6.1.0) [35]. MS/MS spectra were searched against the Human Uniprot protein sequence database (December 2014 with 88,657 entries) using the Andromeda search engine [36]. Primary searches were performed using a 6 ppm precursor ion tolerance for total protein-level analysis. MS/MS ion tolerance was set to 20 ppm. Cysteine carbamidomethylation was set as a fixed modification. Protein N-acetylation and methionine oxidation were considered variable modifications. Enzyme specificity was set to full tryptic digestion. Peptides with a minimum length of six amino acids and up to two missed cleavages were considered. The required FDR was set to 1% at peptide, protein, and modification levels. To maximize the number of quantification events across samples, we enabled the “Match between Runs” option on the MaxQuant platform.

#### 4.2.6. Label-Free Quantification and Statistical Analysis

For label-free quantification, the iBAQ algorithm was used as part of the MaxQuant platform [37]. Briefly, iBAQ values, determined using MaxQuant, were the raw intensities divided by the number of theoretical peptides. Thus, iBAQ values were proportional to molar quantities of the proteins. Perseus software was used for statistical analysis [38]. First, we eliminated proteins identified as “reverse” and “only identified by site”. After filtering values of at least 70% in each group, missing values were imputed using a width of 0.3 and down shift of 1.8. Finally, data were normalized using a width adjustment function, which subtracts the medians and scales all values in a sample to yield equal interquartile ranges (IQRs) [39]. For pairwise proteome comparisons, we performed a two-sided *t*-test with a significance level (*p*-value) of <0.05 and fold-change of >1.5. Support vector machine analysis was performed using the R/Bioconductor package “GNC” [15].

#### 4.2.7. Bioinformatic Analysis

GO enrichment analysis was performed using the DAVID bioinformatics resources (https://david.ncifcrf.gov/). GO-terms and corresponding *p*-values were subsequently submitted to ReViGO [40], and visualized using high semantic similarity-based treemaps. Tumor purity was assessed using the R package “ESTIMATE” on the basis of the expression levels of marker genes in stromal and immune cells [41].

### 4.3. Validation via IHC Analysis

#### 4.3.1. TMA Construction

Prognostic implications of protein biomarkers, identified through proteomic analyses, were validated via IHC staining using a separate dataset consisting of chemotherapy-naïve, FFPE cancer tissues resected from the primary (non-metastatic) ovarian mass intraoperatively during debulking surgery (PDS cases) or diagnostic laparoscopy (NAC cases) (*n* = 107). After tissues were retrieved from the pathology archive of SNUH, they were histologically assessed through hematoxylin and eosin staining. To construct a TMA, three cores (2 mm in diameter) per patient were embedded in new recipient FFPE blocks using a trephine apparatus (Superbiochips Laboratories, Seoul, Korea).

#### 4.3.2. IHC Staining

IHC staining for AAT, NFKB, PMVK, VAP1, FABP4, PF4, APOA1, and AGP was performed using 4 μm thick TMA sections using a Benchmark autostainer (Ventana, Tucson, AZ, USA) in accordance with the manufacturer’s instructions (Table S8).

Because IHC staining of these eight antibodies and its prognostic effects was not previously evaluated in HGSOC, we determined the optimal cutoff values for each IHC staining, based on the sample distribution and prognostic significance (Table S8). Briefly, the extent (0–20%, 20–50%, 50–70%, 70–100%) and intensity (absent, weak, moderate, strong) of cytoplasmic/membranous immunoreactivity were semi-quantitatively assessed. Thereafter, the expression level of each protein was dichotomized into high versus low expression (Figure S4).

### 4.4. Statistical Analysis

Descriptive statistics were used to describe clinicopathologic characteristics of the study population. Patient characteristics were compared between the good and poor prognosis groups, and between groups showing low and high expression of each protein biomarker. We used Student’s *t* and Mann–Whitney U tests to compare continuous variables, and Pearson’s Chi-squared and Fisher’s exact tests to compare categorical variables. Kaplan–Meier methods with log-rank test were used for survival analysis. Multivariate analysis was performed using a Cox proportional-hazards model, and aHRs and 95% CIs were calculated. These analyses were conducted using SPSS software (version 25.0; SPSS Inc., Chicago, IL, USA). All statistical tests were two-sided, and a *p*-value < 0.05 was considered statistically significant.

We constructed regression- and score-based models predicting 18-month PFS using clinical variables and IHC results of 107 patients with primary HGSOC. To evaluate the performance of regression-based predictive models, we performed leave-one-out cross-validation with the consideration of a small sample size. In brief, leave-one-out cross-validation constructs *n* models repetitively, by training the model with *n*-1 samples and testing with the remaining one, where *n* is the sample size. This analysis was repeated for all samples, and *n* predicted values were obtained on the basis of *n* models. We computed AUC using the predicted values and the observed values of the response variable. Finally, we simplified the regression-based model into a score-based model. In this study, each predictor has a single binary value, either 0 or 1. We inverted the original values of the predictors with a negative coefficient (0/1 to 1/0) so that all the direction of effects be positive. Then, a total score for the prediction of 18-month PFS was determined by simply adding all the predictors without coefficient. 

## 5. Conclusions

In conclusion, we successfully generated a proteomic landscape of HGSOC and identified six protein biomarkers to predict the prognosis of HGSOC. These biomarkers are potentially applicable for the development of novel molecular therapeutic agents in the future. Further translational studies and prospective validation studies are warranted to determine the underlying mechanisms of action and interactions among these biomarkers.

## Figures and Tables

**Table 1:** Clinicopathologic characteristics of the patients who underwent prognostic validation.

Characteristics	All (*n* = 107, %)
Age, years	
Mean ± SD	55.6 ± 10.1
Menopause	69 (71.9)
Personal history of breast cancer	16 (15.0)
Family history of breast cancer	4 (3.7)
Family history of ovarian cancer	5 (4.7)
Serum CA-125, IU/ml	
Median (range)	677.5 (5.1–11,630.0)
FIGO stage	
I-II	9 (8.4)
III	68 (63.6)
IV	30 (28.0)
Primary treatment strategy	
PDS	102 (95.3)
NAC	5 (4.7)
Residual tumor after PDS/IDS	
No gross	73 (68.2)
<1 cm	21 (19.6)
≥1 and <2 cm	7 (6.5)
≥2 cm	6 (5.6)
Recurrence	
No	56 (52.3)
Yes	51 (47.7)
No post-operative chemotherapy (within recurrent disease)	1 (0.9)
PSR ^1^ (within recurrent disease)	38 (35.5)
PRR (within recurrent disease)	12 (11.2)
Platinum sensitivity	
Platinum-sensitive ^2^	72 (67.3)
Platinum-resistant	12 (11.2)
Germline *BRCA* mutation	
*BRCA1*	37 (34.6)
*BRCA2*	17 (15.9)
Both	0

^1^ PSR was defined as relapse ≥6 months after completion of taxane- and platinum-based chemotherapy, whereas PRR as relapse <6 months. ^2^ In addition to PSR, the patients who completed taxane- and platinum-based chemotherapy and did not experience disease recurrence during at least six months of follow-up period were considered platinum-sensitive. Abbreviations: CA-125, cancer antigen 125; FIGO, International Federation of Gynecology and Obstetrics; IDS, interval debulking surgery; NAC, neoadjuvant chemotherapy; PDS, primary debulking surgery; PRR, platinum-resistant recurrence; PSR, platinum-sensitive recurrence; SD, standard deviation.

**Table 2:** Factors associated with progression-free survival.

Characteristics	Multivariate Analysis											
	aHR	95% CI	aHR	95% CI	aHR	95% CI	aHR	95% CI	aHR	95% CI	aHR	95% CI
Age, years		*p* = 0.072		*p* = 0.667		*p* = 0.879		*p* = 0.417		*p* = 0.332		*p* = 0.365
≥55 vs. <55	1.755	0.951-3.238	1.133	0.641–2.005	1.046	0.586-1.866	1.265	0.717-2.230	1.327	0.750-2.349	1.300	0.737-2.295
CA-125, IU/ml		*p* = 0.028		*p* = 0.061		*p* = 0.103		*p* = 0.066		*p* = 0.124		*p* = 0.157
≥700 vs. <700	1.911	1.073-3.405	1.720	0.976–3.031	1.603	0.909–2.826	1.695	0.965–2.977	1.553	0.886–2.721	1.500	0.856–2.628
FIGO stage		*p* = 0.470		*p* = 0.182		*p* = 0.260		*p* = 0.304		*p* = 0.281		*p* = 0.255
III–IV vs. I–II	2.149	0.270–17.098	4.010	0.522–30.829	3.220	0.421–24.652	2.920	0.379–22.499	3.066	0.400–23.498	3.227	0.429–24.274
Residual tumor after PDS/IDS		*p* = 0.057		*p* = 0.183		*p* = 0.019		*p* = 0.142		*p* = 0.118		*p* = 0.137
Gross vs. No gross	1.732	0.985–3.048	1.474	0.833–2.608	2.020	1.124–3.630	1.531	0.868–2.703	1.578	0.891–2.794	1.538	0.872–2.711
Germline *BRCA* status		*p* = 0.085		*p* = 0.101		*p* = 0.425		*p* = 0.094		*p* = 0.088		*p* = 0.162
Mutation vs. WT	0.598	0.333–1.073	0.614	0.343–1.099	0.780	0.424–1.436	0.600	0.329–1.091	0.594	0.326–1.081	0.654	0.361–1.186
AAT		*p* = 0.006										
High vs. Low	0.398	0.207–0.768										
NFKB				*p* = 0.030								
High vs. Low			0.424	0.196–0.920								
PMVK						*p* = 0.009						
High vs. Low					0.430	0.228–0.809						
VAP1								*p* = 0.024				
High vs. Low							1.911	1.089–3.354				
FABP4										*p* = 0.023		
High vs. Low									1.908	1.093–3.331		
PF4												*p* = 0.017
High vs. Low											2.071	1.139–3.765

Abbreviations: aHR, adjusted hazard ratio; CI, confidence interval; CA-125, cancer antigen 125; FIGO, International Federation of Gynecology and Obstetrics; IDS, interval debulking surgery; PDS, primary debulking surgery; WT, wild-type; AAT, α1-antitrypsin; NFKB, nuclear factor-κB; PMVK, phosphomevalonate kinase; VAP1, vascular adhesion protein 1; FABP4, fatty acid-binding protein 4; PF4, platelet factor 4.

## Supplementary Materials

The following are available online at https://www.mdpi.com/2072-6694/12/4/790/s1, Figure S1: A comparison of progression-free survival between good and poor prognosis groups; Figure S2: Reproducibility between protein expression values obtained from the good and poor prognosis groups; Figure S3: Assessment of tumor purity in the good and poor prognosis groups; Figure S4: Representative images of high expression and low expression of each protein. (a) AAT; (b) NFKB; (c) PMVK; (d) VAP1; (e) FABP4; (f) PF4; (g) APOA1; (h) AGP; Figure S5: Power analysis of a multivariate model consisting of the six protein biomarkers with variable sample sizes and censoring rates; Figure S6: Flow diagram illustrating the overall study design; Table S1: Clinicopathologic characteristics of the patients who underwent proteomic analysis; Table S2: List of total identified proteins; Table S3: List of differentially expressed proteins between good and poor prognosis groups; Table S4: Results of gene ontology enrichment in differentially expressed proteins; Table S5: Ranked protein list obtained using ”geNetClassifier”; Table S6: Comparisons of patients’ clinicopathologic characteristics by immunohistochemical staining results; Table S7: Comparisons of performance among the developed models predicting 18-month progression-free survival; Table S8: Information on antibodies used in this study and interpretation of results obtained via immunohistochemical staining.
