# Supplementary material for: Proteomic Discovery of Biomarkers to Predict Prognosis of High-Grade Serous Ovarian Carcinoma
Source: Cancers (Basel). 2020 Mar 26;12(4):790. doi: 10.3390/cancers12040790 (PMC7226362; doi:10.3390/cancers12040790)
Supplement: Supplementary file 1 [file cancers-12-00790-s001.zip › Supplementary Table 8.docx]

**Supplementary Table 8.** Information on antibodies used in this study and interpretation of results obtained via immunohistochemical staining

| Marker | Dilution | Catalog No. | High expression cutoff | Assessment of discrepant TMA cores |
| --- | --- | --- | --- | --- |
| AAT | 1:700 | ab9373 | ≥70% of tumor cells showing at least moderate staining intensity | Total tumor cells were measured together |
| NFKB | 1:200 | ab209799 | ≥70% of tumor cells showing strong staining intensity | Two cores meet the cutoff + One core showing moderate intensity in ≥50% of tumor cells is acceptable for high expression |
| PMVK | 1:70 | ab65806 | ≥50% of tumor cells showing at least moderate staining intensity | The majority result was taken |
| VAP1 | 1:100 | ab196739 | ≥20% of tumor cells showing at least moderate staining intensity | The majority result was taken |
| FABP4 | 1:500 | ab13979 | ≥20% of tumor cells showing at least moderate staining intensity | The majority result was taken |
| PF4 | 1:250 | ab49735 | ≥20% of tumor cells showing at least moderate staining intensity | The majority result was taken |
| APOA1 | 1:6000 | ab7613 | ≥20% of tumor cells showing at least moderate staining intensity | The majority result was taken |
| AGP | 1:200 | Ab134042 | ≥20% of tumor cells showing at least moderate staining intensity | The majority result was taken |

Abbreviations: TMA. Tissue microarray; AAT, α1-antitrypsin; NFKB, nuclear factor-κB; PMVK, phosphomevalonate kinase; VAP1, vascular adhesion protein 1; FABP4, fatty acid-binding protein 4 (FABP4); PF4, platelet factor 4; APOA1, apolipoprotein A1; AGP, α1-acid glycoprotein.
