# Supplementary material for: Proteomic Discovery of Biomarkers to Predict Prognosis of High-Grade Serous Ovarian Carcinoma
Source: Cancers (Basel). 2020 Mar 26;12(4):790. doi: 10.3390/cancers12040790 (PMC7226362; doi:10.3390/cancers12040790)
Supplement: Supplementary file 1 [file cancers-12-00790-s001.zip › Supplementary Table 7.docx]

| **Supplementary Table 7.** Comparisons of performance among the developed models predicting 18-month progression-free survival | | | | |  |
| --- | --- | --- | --- | --- | --- |
| Model | Regression-based | | | Score-based | |
|  | Training AUC | Test AUC | AUC | | |
| CA-125, residual tumor ^1^ | 0.640 | 0.437 | 0.647 | | |
| CA-125, residual tumor ^1^, germline *BRCA* status | 0.709 | 0.544 | 0.711 | | |
| CA-125, 6 protein biomarkers ^2^ | 0.863 | 0.772 | 0.829 | | |
| CA-125, residual tumor ^1^, germline *BRCA* status, FIGO stage, 6 protein biomarkers ^2^ | 0.898 | 0.776 | 0.855 | | |
| ^1^ Residual tumor after debulking surgery.  ^2^ Expression levels of AAT, NFKB, PMVK, VAP1, FABP4, and PF4 on immunohistochemical staining of ovarian cancer tissue.  All variables were binarized as follows: CA-125 (≥700 vs. <700 IU/ml); residual tumor (gross vs. no gross); germline *BRCA* mutation (mutation vs. wild-type); FIGO stage (III-IV vs. I-II); 6 protein biomarkers (high expression vs. low expression).  Abbreviations: AUC, area under the receiver operating characteristic curve; CA-125, cancer antigen 125; FIGO, International Federation of Gynecology and Obstetrics. | | | | |  |
