# Supplementary material for: Proteomic Discovery of Biomarkers to Predict Prognosis of High-Grade Serous Ovarian Carcinoma
Source: Cancers (Basel). 2020 Mar 26;12(4):790. doi: 10.3390/cancers12040790 (PMC7226362; doi:10.3390/cancers12040790)
Supplement: Supplementary file 1 [file cancers-12-00790-s001.zip › Supplementary Table 6.docx]

**Supplementary Table 6.** Comparisons of patients’ clinicopathologic characteristics by immunohistochemical staining results

|  | AAT expression | | | NFKB expression | | | PMVK expression | | | VAP1 expression | | | FABP4 expression | | | PF4 expression | | |
| --- | --- | --- | --- | --- | --- | --- | --- | --- | --- | --- | --- | --- | --- | --- | --- | --- | --- | --- |
|  | Low  (*n*=50, %) | High  (*n*=57, %) | *P* | Low  (*n*=77, %) | High  (*n*=30, %) | *P* | Low  (*n*=52, %) | High  (*n*=55, %) | *P* | Low  (*n*=77, %) | High  (*n*=30, %) | *P* | Low  (*n*=71, %) | High  (*n*=36, %) | *p* | Low  (*n*=81, %) | High  (*n*=26, %) | *P* |
| Age, years |  |  | 0.420 |  |  | 0.377 |  |  | 0.137 |  |  | 0.201 |  |  | 0.789 |  |  | >0.999 |
| ≥55 | 25 (50.0) | 34 (59.6) |  | 45 (58.4) | 14 (46.7) |  | 33 (63.5) | 26 (47.3) |  | 39 (50.6) | 20 (66.7) |  | 38 (53.5) | 21 (58.3) |  | 45 (55.6) | 14 (53.8) |  |
| <55 | 25 (50.0) | 23 (40.4) |  | 32 (41.6) | 16 (53.3) |  | 19 (36.5) | 29 (52.7) |  | 38 (49.4) | 10 (33.3) |  | 33 (46.5) | 15 (41.7) |  | 36 (44.4) | 12 (46.2) |  |
| CA-125, IU/ml |  |  | 0.373 |  |  | 0.803 |  |  | 0.291 |  |  | 0.496 |  |  | 0.540 |  |  | 0.869 |
| ≥700 | 22 (44.0) | 30 (52.6) |  | 38 (4.4) | 14 (46.7) |  | 28 (53.8) | 24 (43.6) |  | 39 (50.6) | 13 (43.3) |  | 36 (50.7) | 16 (44.4) |  | 39 (48.1) | 13 (50.0) |  |
| <700 | 28 (56.0) | 27 (47.4) |  | 39 (50.6) | 16 (53.3) |  | 24 (46.2) | 31 (56.4) |  | 38 (49.4) | 17 (56.7) |  | 35 (49.3) | 20 (55.6) |  | 42 (51.9) | 13 (50.0) |  |
| FIGO stage |  |  | 0.170 |  |  | 0.113 |  |  | 0.490 |  |  | 0.440 |  |  | >0.999 |  |  | 0.216 |
| III-IV | 48 (96.0) | 50 (87.7) |  | 73 (94.8) | 25 (83.3) |  | 49 (94.2) | 49 (89.1) |  | 69 (89.6) | 29 (96.7) |  | 65 (91.5) | 33 (91.7) |  | 76 (93.8) | 22 (84.6) |  |
| I-II | 2 (4.0) | 7 (12.3) |  | 4 (5.2) | 5 (16.7) |  | 3 (5.8) | 6 (10.9) |  | 8 (10.4) | 1 (3.3) |  | 6 (8.5) | 3 (8.3) |  | 5 (6.2) | 4 (15.4) |  |
| Residual tumor  after PDS/IDS |  |  | 0.564 |  |  | 0.020 |  |  | 0.992 |  |  | >0.999 |  |  | 0.641 |  |  | 0.908 |
| Gross | 14 (28.0) | 20 (35.1) |  | 30 (39.0) | 4 (13.3) |  | 16 (30.8) | 18 (32.7) |  | 24 (31.2) | 10 (33.3) |  | 21 (29.6) | 13 (36.1) |  | 25 (30.9) | 9 (34.6) |  |
| No gross | 36 (72.0) | 37 (64.9) |  | 47 (61.0) | 26 (86.7) |  | 36 (69.2) | 37 (67.3) |  | 53 (68.8) | 20 (66.7) |  | 50 (70.4) | 23 (63.9) |  | 56 (69.1) | 17 (65.4) |  |
| *BRCA* status |  |  | 0.776 |  |  | 0.877 |  |  | 0.067 |  |  | 0.783 |  |  | >0.999 |  |  | 0.237 |
| Mutation | 24 (48.0) | 30 (52.6) |  | 38 (49.4) | 16 (53.3) |  | 21 (40.4) | 33 (60.0) |  | 40 (51.9) | 14 (46.7) |  | 36 (50.7) | 18 (50.0) |  | 44 (54.3) | 10 (38.5) |  |
| Wild-type | 26 (52.0) | 27 (47.4) |  | 39 (50.6) | 14 (46.7) |  | 31 (59.6) | 22 (40.0) |  | 37 (48.1) | 16 (53.3) |  | 35 (49.3) | 18 (50.0) |  | 37 (45.7) | 16 (61.5) |  |
| Platinum sensitivity^*^ |  |  | 0.397 |  |  | 0.166 |  |  | 0.023 |  |  | 0.098 |  |  | 0.203 |  |  | 0.065 |
| Resistant | 8 (18.6) | 4 (9.8) |  | 11 (18.0) | 1 (4.3) |  | 10 (24.4) | 2 (4.7) |  | 5 (9.1) | 7 (24.1) |  | 5 (9.8) | 7 (21.2) |  | 6 (9.5) | 6 (28.6) |  |
| Sensitive | 35 (81.4) | 37 (90.2) |  | 50 (82.0) | 22 (95.7) |  | 31 (75.6) | 41 (95.3) |  | 50 (90.1) | 22 (75.9) |  | 46 (90.2) | 26 (78.8) |  | 57 (90.5) | 15 (71.4) |  |

Abbreviations: AAT, α1-antitrypsin; NFKB, nuclear factor-κB; PMVK, phosphomevalonate kinase; VAP1, vascular adhesion protein 1; FABP4, fatty acid-binding protein 4 (FABP4); PF4, platelet factor 4; CA-125, cancer antigen 125; FIGO, International Federation of Gynecology and Obstetrics; PDS, primary debulking surgery; IDS, interval debulking surgery.

^*^Assessed in 84 patients
