# Supplementary material for: Proteomic Discovery of Biomarkers to Predict Prognosis of High-Grade Serous Ovarian Carcinoma
Source: Cancers (Basel). 2020 Mar 26;12(4):790. doi: 10.3390/cancers12040790 (PMC7226362; doi:10.3390/cancers12040790)
Supplement: Supplementary file 1 [file cancers-12-00790-s001.zip › Supplementary Table 1.docx]

**Supplementary Table 1.** Clinicopathologic characteristics of the patients who underwent proteomic analysis

| Characteristics | All  (*n*=12, %) | Good prognosis  group  (*n*=6, %) | Poor prognosis  group  (*n*=6, %) | *P* |
| --- | --- | --- | --- | --- |
| Age, years |  |  |  |  |
| Mean ± SD | 56.5 ± 10.3 | 51.9 ± 7.2 | 61.1 ± 11.4 | 0.126 |
| Menopause | 8 (66.7) | 4 (66.7) | 4 (66.7) | >0.999 |
| Family history of breast cancer | 1 (8.3) | 1 (16.7) | 0 | >0.999 |
| Family history of ovarian cancer | 0 | 0 | 0 | N/A |
| Serum CA-125, IU/ml |  |  |  |  |
| Median (range) | 619.0 (5.1-3780.0) | 509.5 (5.1-3545.0) | 869.0 (248.9-3780.0) | 0.631 |
| FIGO stage |  |  |  | 0.545 |
| III | 8 (66.7) | 5 (83.3) | 3 (50.0) |  |
| IV | 4 (33.3) | 1 (16.7) | 3 (50.0) |  |
| Residual tumor after PDS |  |  |  | >0.999 |
| No gross | 9 (75.0) | 5 (83.3) | 4 (66.7) |  |
| <1 cm | 3 (25.0) | 1 (16.7) | 2 (33.3) |  |
| Recurrence |  |  |  |  |
| No | 1 (8.3) | 1 (16.7) | 0 | >0.999 |
| Yes | 11 (91.7) | 5 (83.3) | 6 (100.0) |  |
| PSR^*^ | 9 (75.0) | 5 (83.3) | 4 (66.7) | 0.455 |
| PRR | 2 (16.7) | 0 | 2 (33.3) |  |
| Platinum sensitivity |  |  |  | 0.455 |
| Platinum-sensitive^†^ | 10 (83.3) | 6 (100.0) | 4 (66.7) |  |
| Platinum-resistant | 2 (16.7) | 0 | 2 (33.3) |  |
| *BRCA* mutation |  |  |  | 0.002 |
| *BRCA1* | 4 (33.3) | 4 (66.7) | 0 |  |
| *BRCA2* | 2 (16.7) | 2 (33.3) | 0 |  |
| Both | 0 | 0 | 0 |  |
| Abbreviations: CA-125, cancer antigen 125; FIGO, International Federation of Gynecology and Obstetrics; N/A, not applicable; PDS, primary debulking surgery; PRR, platinum-resistant recurrence; PSR, platinum-sensitive recurrence; SD, standard deviation.  ^*^PSR was defined as relapse ≥6 months after completion of taxane- and platinum-based chemotherapy, whereas PRR as relapse <6 months.  ^†^In addition to PSR, the patients who completed taxane- and platinum-based chemotherapy and did not experience disease recurrence during at least 6 months of follow-up period were considered platinum-sensitive. | | | | |
