# Supplementary figures and images for: Proteomic Discovery of Biomarkers to Predict Prognosis of High-Grade Serous Ovarian Carcinoma
Source: Cancers (Basel). 2020 Mar 26;12(4):790. doi: 10.3390/cancers12040790 (PMC7226362; doi:10.3390/cancers12040790)

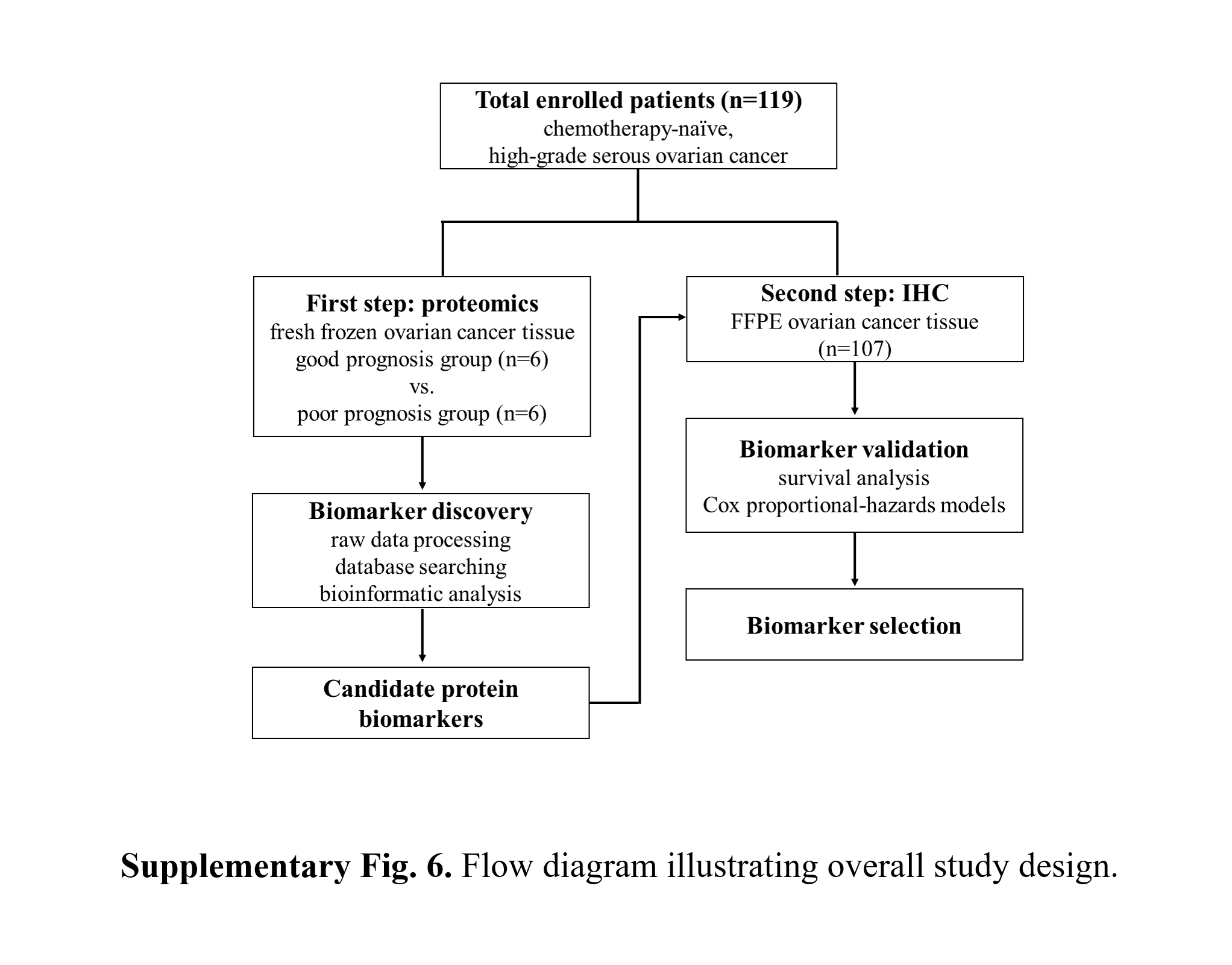

Supplement: Supplementary file 1 [file cancers-12-00790-s001.zip › Supplementary Fig. 6.tif]

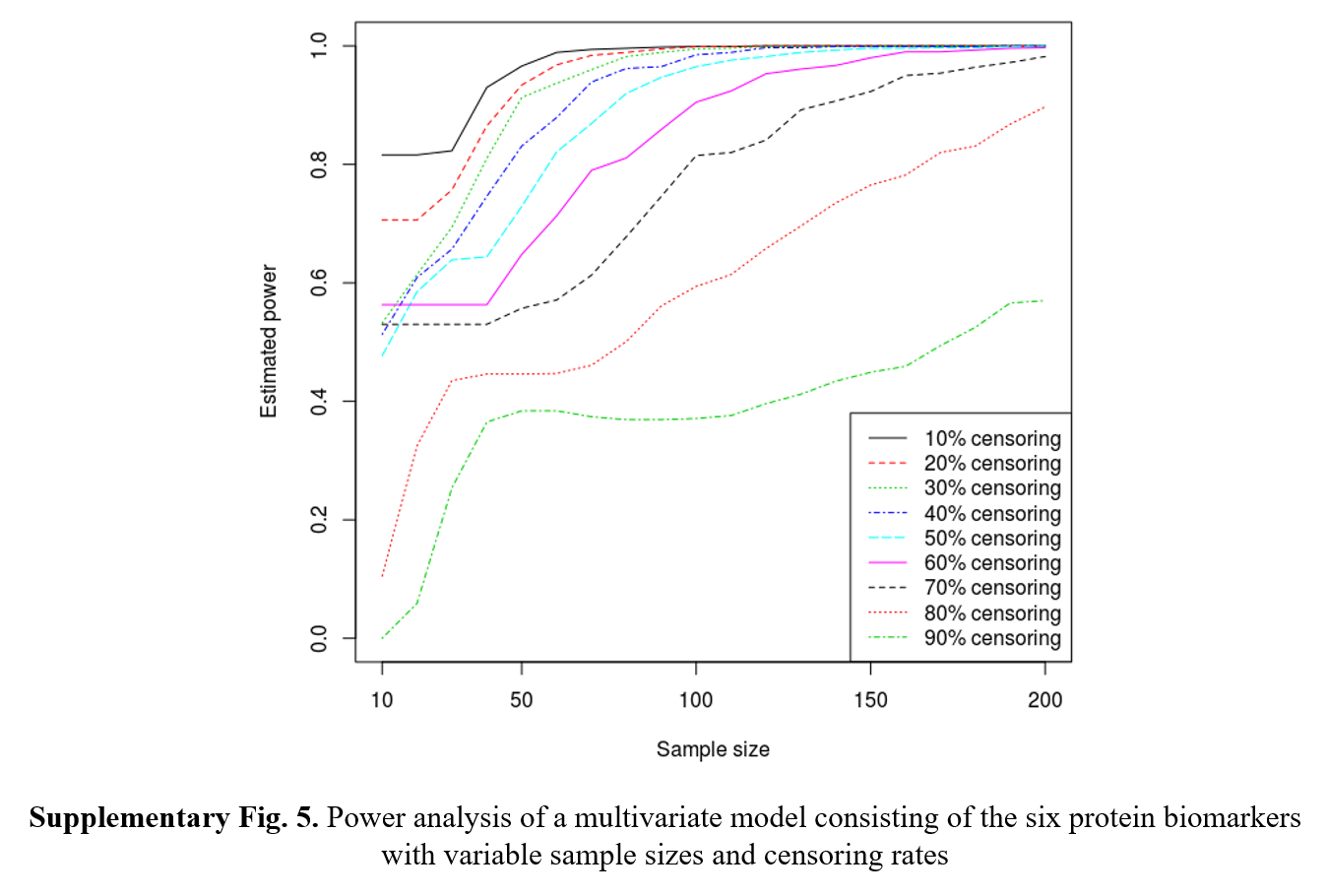

Supplement: Supplementary file 1 [file cancers-12-00790-s001.zip › Supplementary Fig. 5.tif]

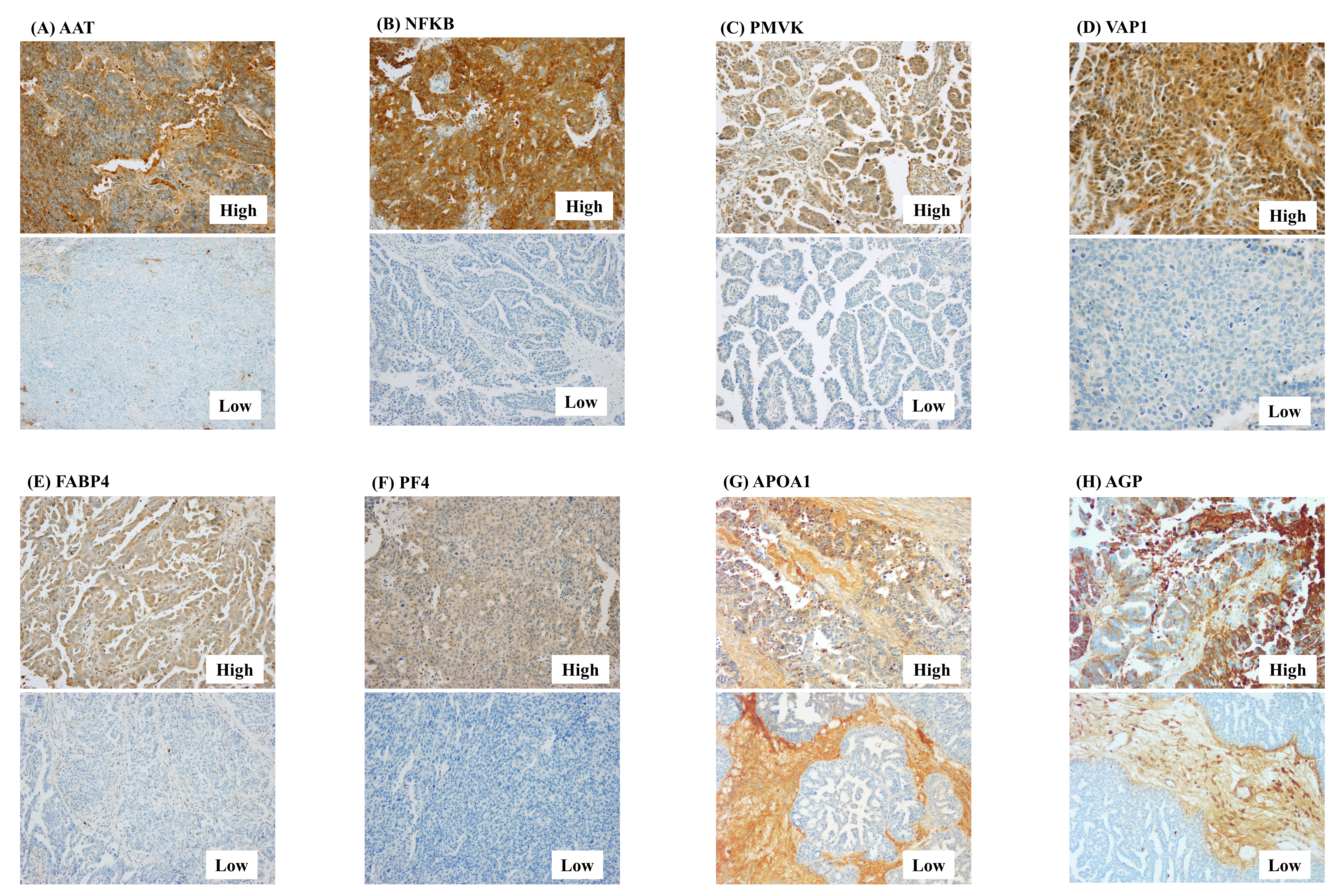

Supplement: Supplementary file 1 [file cancers-12-00790-s001.zip › Supplementary Fig. 4.tiff]

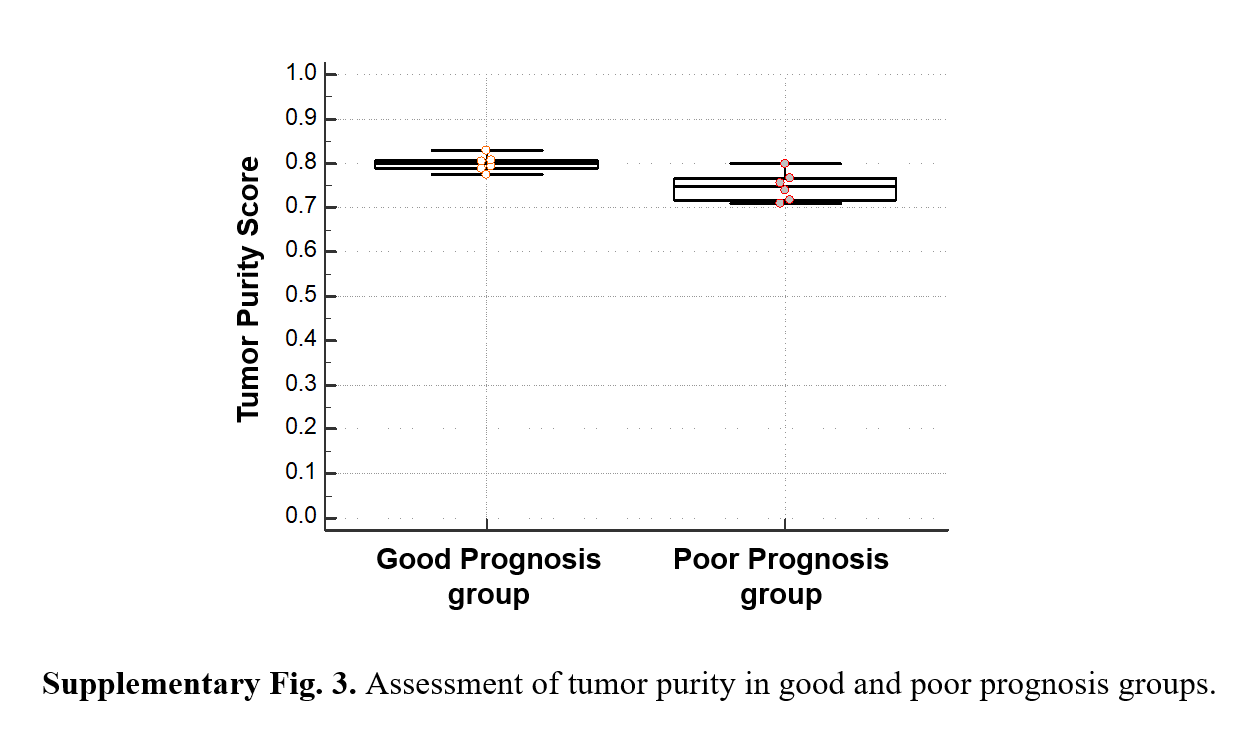

Supplement: Supplementary file 1 [file cancers-12-00790-s001.zip › Supplementary Fig. 3.tif]

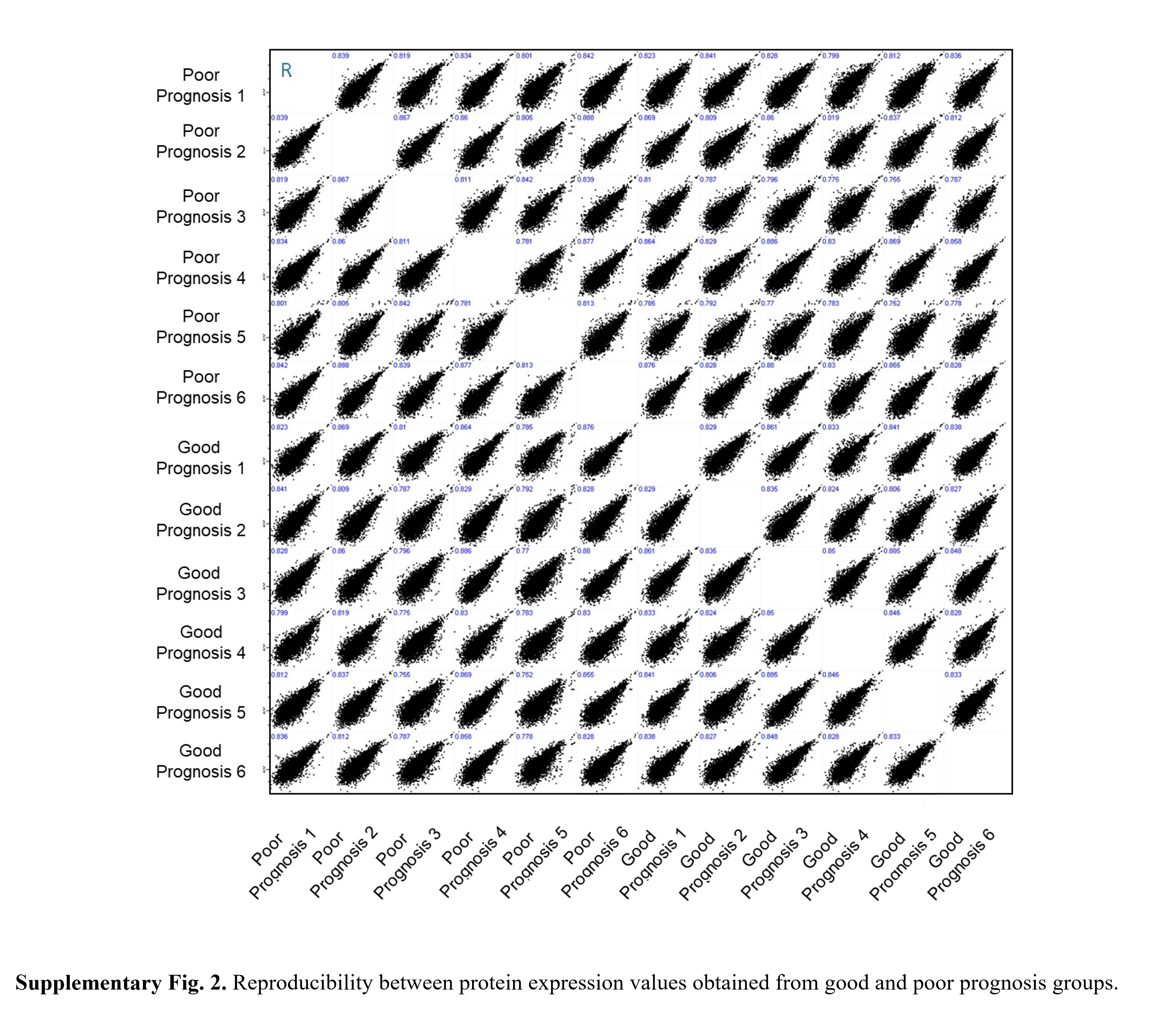

Supplement: Supplementary file 1 [file cancers-12-00790-s001.zip › Supplementary Fig. 2.png]

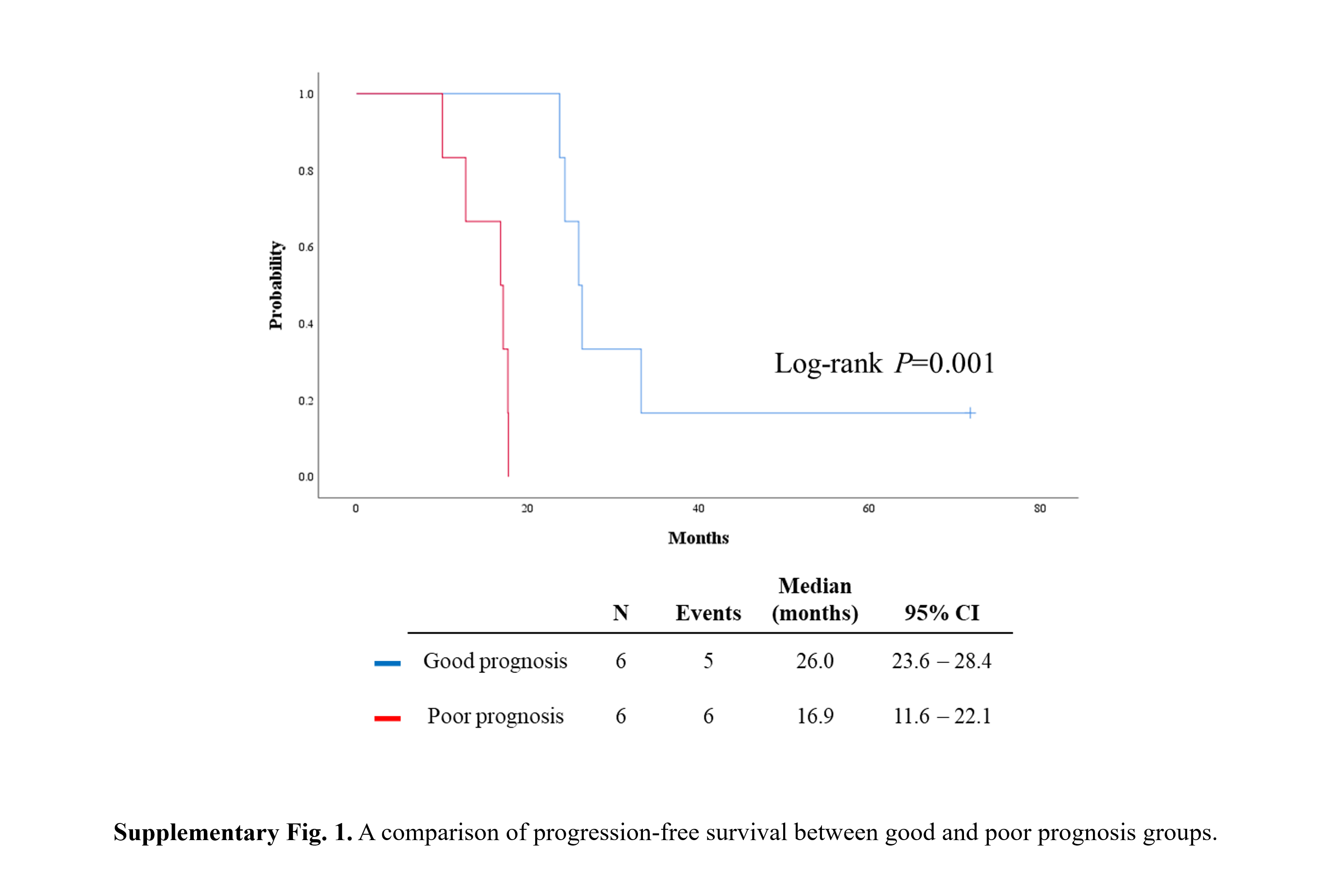

Supplement: Supplementary file 1 [file cancers-12-00790-s001.zip › Supplementary Fig. 1.tif]
